# Design of a randomised, double-blind, crossover, placebo-controlled trial of effects of sildenafil on cerebrovascular function in small vessel disease: Oxford haemodynamic adaptation to reduce pulsatility trial (OxHARP)

**DOI:** 10.1177/23969873211026698

**Published:** 2021-06-23

**Authors:** Alastair Webb, David Werring, Jesse Dawson, Alex Rothman, Amy Lawson, Karolina Wartolowska

**Affiliations:** 1 Wolfson Centre for Prevention of Stroke and Dementia, University of Oxford, Oxford, UK; 2 Stroke Research Centre, UCL Institute of Neurology, London, UK; 3 Institute of Cardiovascular and Medical Sciences, College of Medical, Veterinary & Life Sciences, Queen Elizabeth University Hospital, University of Glasgow, Glasgow, UK; 4 Infection, Immunity and Cardiovascular Disease, University of Sheffield, Sheffield, UK

**Keywords:** Vasodilator, protocol, cerebral pulsatility, cerebral reactivity, small vessel disease

## Abstract

**Background:**

Cerebral small vessel disease (SVD) is associated with increased cerebrovascular pulsatility, endothelial dysfunction, and impaired vascular reactivity. Vasodilating phosphodiesterase inhibitors may improve cardiovascular pulsatility and reactivity, and potentially reduce progression of SVD.

Hypothesis: Sildenafil, a PDE5 inhibitor, will reduce cerebrovascular pulsatility and increase cerebrovascular reactivity compared to placebo, and is non-inferior to cilostazol, a PDE3 inhibitor.

**Methods:**

OxHARP is a randomised, double-blind, crossover trial of sildenafil 50 mg thrice daily, cilostazol 100 mg twice daily and placebo in 75 patients with mild to moderate small vessel disease and a previous lacunar or cryptogenic stroke or TIA. Participants undergo a physiological assessment at baseline and on each treatment, including transcranial Doppler ultrasound (TCD, DWL DopplerBox) to assess cerebrovascular pulsatility and reactivity to 4–6% carbon dioxide. In up to 60 patients, cerebrovascular pulsatility, perfusion and reactivity will also be assessed by MRI.

**Outcome measures:**

The primary outcome is difference in middle cerebral artery pulsatility (Gosling’s Pulsatility Index, PI) after 3 weeks of sildenafil versus placebo. Secondary outcomes including non-inferiority of sildenafil vs cilostazol in effects on PI, percentage increase in MCA blood flow velocity and BOLD-fMRI response during inhalation of 4–6% carbon dioxide.

**Discussion:**

Reduction in cerebral pulsatility and increased cerebrovascular reactivity during treatment with sildenafil would indicate potential benefit to prevent progression of SVD, suggesting a need for trials with clinical outcomes.

Trial Registration OxHARP is registered with ClinicalTrials.org, NCT03855332

## Introduction

Chronic injury to the small vessels of the brain (‘small vessel disease’) is associated with acute lacunar stroke,^1^ progressive cognitive decline,^2^ late-onset refractory depression,^3^ functional impairment in daily living^4^ and increased mortality.^5^ Despite accounting for approximately 30% of strokes and 40% of dementia,^6^ the underlying mechanism of small vessel injury is unclear. White matter hyperintensities are highly prevalent in the population, affecting the majority of patients over the age of 65.^7^ However, even patients with advanced imaging changes can remain functionally independent,^7^ indicating a pre-clinical stage where intervention may prevent progression of SVD and resulting morbidity. Development of treatments to prevent progression of SVD, particularly in ‘at risk’ patients with non-embolic strokes, is vital to reduce the resulting morbidity in the population.

Hypertension is the strongest modifiable risk factor for small vessel disease, but there is only limited evidence that reduction of blood pressure alone significantly reduces progression of SVD.^8^ Pulsatility of blood flow to the brain is associated with small vessel disease,^9^ resulting from increased transmission of the pulsatile aortic waveform to the brain through stiff conduit vessels. Secondly, small vessel disease is associated with endothelial dysfunction, demonstrated by impaired cerebrovascular reactivity and breakdown of the blood-brain barrier.^10^ This may be secondary to hypertension or arterial pulsatility, but it may also reflect a local primary endotheliopathy.

Aortic pulsatility, aortic stiffness and transmission of the aortic waveform are increased by reflection of the outgoing aortic pressure wave from the systemic small vessels back towards the aorta, increasing aortic pulsatility. The resulting enhanced pulsatility reaching the low resistance small vessels in the brain may cause increased shear stress during systole and potential hypoperfusion of tissues during diastole.^11^ Increased aortic pulsatility may be modifiable by vasodilating medications that delay the site and severity of wave reflection. Such medications may also act upon muscular conduit vessels (distal internal carotid or middle cerebral arteries (MCA)) to increase elasticity, improve dampening of the aortic waveform and reducing pulsatility at distal vessels. Phosphodiesterase inhibitors such as sildenafil (a PDE5 inhibitor) and cilostazol (a PDE3 inhibitor) enhance the cGMP pathways downstream of nitric oxide-dependent endothelial signalling, potentially reducing wave reflection and enhancing cerebrovascular reactivity, but the effect of sildenafil on the intracranial vessels and brain vasculature has not been adequately assessed. Cilostazol has been shown to reduce cerebral pulsatility and reduce the risk of stroke,^12^ but its additional antiplatelet effects complicate how it could be used in conjunction with current antiplatelet strategies. We therefore aim to test the effect of sildenafil on cerebral pulsatility assessed by transcranial ultrasound (TCD) and reactivity compared to placebo, and its non-inferiority compared to cilostazol.

## Methods

### Study design

**Figure 1.**
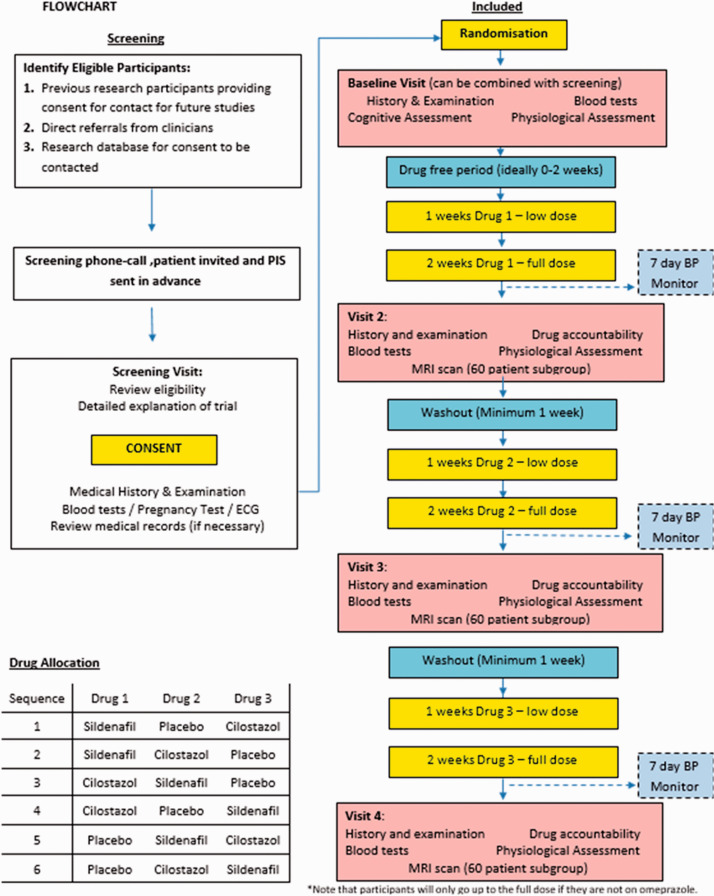
Flowchart of each patient’s progress through the study.

OxHARP is a double-blind, randomised, placebo-controlled, crossover study with physiological endpoints. Participants receive placebo, sildenafil and cilostazol for three weeks each in random sequence, with a minimum 1 week washout period between treatments, and with a dose-titration step at 1 week (see Figure 1). To maintain blinding, all medications are overencapsulated, and administered three times daily, with a placebo dose at mid-day during cilostazol treatment. On the final day of treatment, participants undergo a physiological assessment to determine cerebral pulsatility and cerebrovascular reactivity, with further physiological tests to assess potential mechanisms and correlated physiological effects. A subset of up to 60 patients may undergo an MRI scan whilst on sildenafil and placebo, with up to 30 imaged on all three treatments.

### Trial status

The first patient was included on 11^th^ July 2019, with 15 patients recruited by 31^st^ January 2020, including 13 participants recruited to the MRI substudy, and full recruitment initially expected by December 2022. Due to COVID-19, the study was halted in March 2020, with resumption of recruitment in September 2020, with 27 patients recruited by December 2020. As a result, full recruitment is now planned by December 2023, allowing for further disruption to recruitment.

### Ethical and regulatory approval

OxHARP is sponsored by the University of Oxford, approved by the UK Health Research Authority and South Central – Oxford C Research Ethics Committee (19/SC/0022), and is registered with ClinicalTrials.org (NCT03855332).

### Population

75 patients with a history of a stroke or probable TIA more than 1 month previously, of cryptogenic or lacunar aetiology, mild to moderate SVD evident on brain imaging within the past 6 years and adequate temporal bone windows.

### Patient identification and recruitment

Potential participants are approached by their clinical teams during an acute inpatient stay, at a TIA clinic or at a follow-up clinic at the core site (Oxford) or regional patient identification centres (High Wycombe, Swindon, Reading), or may be approached directly following participation in other research studies or from approved registries of patients. At the first visit, participants are reviewed face-to-face by a study physician, eligibility confirmed and full informed consent obtained.

### Participant schedule

The study comprises 4 visits over a minimum of 11 weeks, including a baseline/screening visit and 3 ‘on-treatment’ visits, separated by a minimum of 1 week washout and 3 weeks of treatment (1 week half-dose, 2 weeks full dose) with each investigational agent in randomised order (Figure 1). At the screening or baseline visit, participants give full informed consent, a medical history and physical examination, cognitive assessment (Montreal Cognitive Assessment, Digit Symbol Test, Fluid Intelligence), blood tests, ECG and the core physiological assessment. At each subsequent visit, participants have a clinical assessment, blood tests and take their final treatment dose 30 minutes prior to the core physiological assessment. Patients participating in the MRI substudy undergo an MRI scan following the core physiological assessment. Participants take their blood pressure at home (twice daily, morning and evening, three readings each time) during the week preceding each follow-up visit (see Table 1).

**Table 1.** Study schedule.

Activity/assessment	Screening–21 to 0	BaselineDay 0	Visit 1≥Day 28	Visit 2≥Day 56	Visit 3≥Day 84
Informed consent	X				
Eligibility criteria	X				
Demographics	X				
Medical/history	X	X			
Vital signs	X		X	X	X
Physical exam	X		X	X	X
Cognitive assessment		X			
Pregnancy test	(X)	(X)	(X)	(X)	(X)
Laboratory tests	X	X	X	X	X
ECG	X				
Randomisation		X			
7 days home BP monitoring twice daily for 1 week		X	X	X	X
Study drug dispensation		X	X	X	
mRS (disability)		X			
NIHSS (stroke severity) only for stroke patients		X			
Drug accountability			X	X	X
Concomitant medication	X	X	X	X	X
Adverse events (AE)			X	X	X
Pulse wave velocity/pulse wave analysis		X	X	X	X
Transcranial (TCD) ultrasound scan		X	X	X	X
Beat-to-beat BP monitoring		X	X	X	X
Peripheral vascular reactivity		X	X	X	X
MRI assessment (up to 60 patients)			X	X	X

### Randomisation

For eligible patients (see Table 2), sequential study IDs are randomly allocated to six treatment schedules (Figure 1), stratified by allocation to the MRI substudy, at the point of production of treatment packs by Huddersfield Pharmacy Manufacturing Unit, and dispensed by the OUH Clinical Trial Pharmacy. Study personnel and participants remain blinded to treatment.

**Table 2.** Inclusion and exclusion criteria.

Inclusion criteria	Exclusion criteria
• Willing and able to give informed consent• Male or Female, aged 18 years or above.• MCA flow recordable on at least one side • Non-disabling, ischaemic stroke or TIA,• >1 month prior to randomisation,• either cryptogenic or lacunar aetiology, confirmed clinically or on brain imaging • White matter hyperintensities on MRI consistent with cerebral small vessel disease:○ Age <60:○ MRI – Fazekas score 1–3 (max 2 points in periventricular or deep score)○ CT – Blennow score 1–3 (max 2 points in periventricular or deep score)○ Age ○ >60: MRI - Fazekas score 1–4 (max 2 points in periventricular or deep score) ○ CT – Blennow score 1–4 (max 2 points in periventricular or deep score)	• Pregnant or breastfeeding women, • Women of childbearing age not taking contraception. • Other major neurological or psychiatric conditions interfering with the study design (e.g. multiple sclerosis)• Other causes of stroke such as○ >50% luminal stenosis (NASCET) ○ Major-risk cardioembolic source of embolism ○ other specific causes of stroke (e.g. arteritis, dissection) • Large vessel occlusion on MRA or CTA • Modified Rankin Score >3 (requires assistance to walk)• Unable to swallow• Renal impairment (eGFR <35ml/min) • Significant biochemical abnormalities (sodium <130, K+ <2.5 or >5.5, LFTs >3 x upper limit of normal range)• Life expectancy <2 years• Contraindication to active agents:○ Concurrent use of alphablocker, nitrates, ketoconazole, erythromycin, anticoagulants or > 1 antiplatelet.○ Heart failure (NYHA 2–4), severe aortic stenosis, unstable angina, myocardial infarction within 6 months, uncontrolled arrhythmias, haemodynamically significant aortic/mitral valve disease○ Previous priapism, anatomical deformation of the penis○ History of non-arteritic ischaemic optic neuropathy○ Bilateral renal artery stenosis, Sickle cell disease, myeloma, leukaemia○ Hypotension (BP <90/60) or uncontrolled hypertension (BP >180/110 despite treatment with 3 antihypertensives)• Scheduled elective surgery or other procedures requiring general anaesthesia during the study.• Participants participating in another research study involving an investigational product in the past 12 weeks.• Predisposition to intracerebral haemorrhage (previous ICH, likely cerebral amyloid angiopathy) or intraocular haemorrhage (uncontrolled diabetic retinopathy or neovascularisation)

### Intervention

25 mg oral sildenafil is taken three times daily for one week, increased to 50 mg three times daily for a further 2 weeks, if tolerated and limiting side-effects do not occur at the higher dose. The principal comparator is placebo, three times daily. The secondary comparator is 50 mg cilostazol twice daily for one week, titrated to 100 mg for 2 further weeks, with a matched placebo at midday.

### Blinding

Participants, the study team and endpoint assessors are blinded to treatment. All medications are overencapsulated and dispensed in identical, scheduled treatment packs. In patients imaged only on sildenafil and placebo, the study team is not blinded to the cilostazol arm, but remains blinded for sildenafil and placebo treatment periods, whilst participants will remain blinded to all treatments. If needed, the chief investigator (AW) will determine if there is clinical indication for code-breaking. Increased erections in response to sildenafil may result in incomplete blinding in men, which will be assessed by participant reporting of any change in sexual behaviour or frequency of erections.

### Physiological assessment

#### Clinical tests

At each visit, all patients undergo a clinical assessment, examination and routine blood tests including full blood count, urea and electrolytes, liver function test and CRP, with BNP or NT-proBNP, gamma-GT, lipid profile, and HbA1C at baseline (Table 1). In women of child-bearing potential, a pregnancy test is performed at the first visit. If an ECG within the previous year is not available, or the medical history indicates that a cardiac event may have occurred since the last ECG, this is performed prior to randomisation. Participants take their last medication dose at the start of each visit, 45 minutes prior to primary outcome measurement.

### Physiological assessment

Middle cerebral artery flow velocity is assessed with DiaMon 2 MHz probes (DWL), as the highest velocity waveform closest to a 50 mm depth, via the transtemporal window. The basilar artery is insonated via the suboccipital window, at the maximum velocity identifiable at the depth closest to 80 mm. Gosling’s Pulsatility Index is derived: (peak flow velocity – trough flow velocity)/mean flow velocity. Where bone windows are adequate, bilateral monitoring of the middle cerebral artery is established with 2 probes held by a comfortable headset, acquired over 10 minutes with concurrent ECG, non-invasive blood pressure monitoring (FMS, Finometer Midi) and end-tidal carbon dioxide monitoring (etCO2, AD Instruments Gas Analyser ML206), via an AD Instruments Powerlab 8/35.^9,13^ Reactivity to CO2 is assessed by 30 seconds of hyperventilation, followed by 2-minute alternating periods of inhalation of medical air and then 4% and 6% CO2, delivered via a respiratory circuit with a well-sealed, non-invasive ventilation mask.

Additional explanatory physiological measures include arterial stiffness by carotid-femoral pulse wave velocity, aortic blood pressure (Sphygmocor, At-Cor Medical, Sydney, Australia) and measures of cerebral autoregulation derived from the 10 minutes of concurrent monitoring of blood pressure and cerebral blood flow velocity (autoregulation index,^14^ transfer function analysis^15^) Peripheral vascular reactivity is assessed by flow mediated slowing (Vicorder, Skidmore Medical, UK), where pulse wave velocity between two brachial sites is determined before and after 5 minutes of supra-systolic occlusion.^16^

### MRI assessment

Up to 60 consenting participants will be scanned on a 3-Tesla Siemens Prisma scanner (Oxford Wellcome Centre for Integrative Neuroimaging (WIN)) at the end of their sildenafil or placebo visits. Participants will lie supine with foam pads, blankets and earplugs to ensure comfort. During sequences that do not require participants attention, they will be able to watch a film using prism glasses. The principal MRI outcome will be change in BOLD-fMRI signal during inhalation of 6% CO2 in medical air, for 2 × 2 minutes periods versus 2-minute periods of medical air (TR 800 ms, TE 30 ms, multi-band acceleration factor 6, flip angle 50 degrees, 66 slices, in-plane resolution 2.4x2.4 mm, slice thickness 2.4 mm).^17^ The secondary MRI outcome is cerebral arterial pulsatility during high-frequency, multi-band BOLD-FMRI (TR 400 ms, TE 22 ms, multi-band acceleration factor 6, flip angle 90°, 30 slices, in-plane resolution 2.9x2.9 mm, slice thickness 3 mm).^18,19^

Participants undergo perfusion imaging at each visit by pseudo-continuous Arterial Spin Labelling (pcASL) sequence (TR 5100 ms, TE 14 ms, flip angle 90 degrees, bolus duration 1800 ms, post-labelling delay times: 300 ms, 600 ms, 900 ms, 1200 ms, 1500 ms, 1800 ms, 2100 ms, 24 slices, slice in-plane resolution 3.4x3.4 mm, slice thickness 4.5 mm). Participants also undergo structural imaging, split across the two visits: T1-weighted scan for registration and volumetric assessment (MPRAGE sequence; TR 2500 ms, TE 4.37, voxel dimensions 1 mm isometric; analysis by FSL VBM); a T2-weighted-Fluid-Attenuated Inversion Recovery scan for assessment of volume of white matter hyperintensities (FLAIR: TR 5000 ms, TE 397 ms, voxel dimensions 1x1x1.1 mm; analysis by BIANCA)^20^; susceptibility weighted imaging (SWI: TR 27 ms, TE1 9.42 ms, TE2 19.7 ms, in-plane resolution 0.8x0.8 mm, and slice thickness 3 mm) to quantify microhaemorrhages; and diffusion tensor imaging (DTI; TR 3600 ms, TE 92 ms, 2x2x2mm voxel) scan, to assess white matter microstructural integrity.

During pulsatility imaging, participants undergo monitoring of pulse oximetry, respiratory bellows, end-tidal CO2 via nasal cannulae (AD Instruments Gas Analyser ML206) and continuous non-invasive blood pressure by bilateral brachial cuffs.^18^ During CO2-reactivity imaging, gas is delivered by a respiratory circuit with sealed, non-invasive ventilation mask and with end-tidal CO2 via a CO2 sampling line.^21^ Each MRI session lasts approximately 50 minutes.

BOLD and ASL will be pre-processed prior to statistical analysis with motion correction (MCFLIRT), B0 unwarping (BBR) and removal of extra-cerebral tissue. Data will undergo spatial and temporal smoothing, where appropriate. Perfusion on ASL will be quantified by Bayesian Inference for Arterial Spin Labelling (BASIL).

### Statistical analysis plan

#### Primary outcome

The primary outcome is difference in Gosling’s Pulsatility Index on sildenafil versus placebo by paired T-test, on the side with a better-quality recording as defined by a blinded assessor.

#### Secondary outcome

Non-inferiority of effects of sildenafil versus cilastazol will be determined from the upper limit of the 95% confidence interval, compared to a 0.08 unit change in MCA-PI.

Differences in reactivity to CO2 (percentage change in mean flow velocity per percentage change in etCO2) and MCA-PI on each treatment (sildenafil, cilostazol, placebo) will be compared by mixed-effect general linear models. Reactivity to CO2 on MRI will be determined individually by general linear models on a voxel-wise basis (FEAT, FSL) for change in BOLD signal per change in end-tidal CO2, stratified by tissue-type, and compared between drug states in a general linear model adjusting for within subject comparisons (FSL FLAME), to test for an interaction between drug allocation and CO2 response.

#### Exploratory outcomes

Effects of treatment on physiological indices will be determined to assess mechanisms of effects on pulsatility and reactivity by mixed-effect, linear models across treatment phases for within-subject comparisons, adjusted for repeated measures. Physiological indices include home systolic and diastolic blood pressure; carotid femoral pulse wave velocity; aortic systolic blood pressure and pulse pressure; beat-to-beat systolic and diastolic blood pressure variability (coefficient of variation: standard deviation/mean); peripheral flow mediated slowing^16^; measures of resting state cerebral autoregulation in both time domain (Mx, Dx, Sx) and frequency domains (transfer function analysis^15^) Interactions with concurrent treatment with vasodilating antihypertensives (ie amlodipine) will be assessed.

Higher level MRI analyses will be performed by general linear models, modelling mean effect for each treatment, differential effect associated with period, and differential effects associated with subject mean. An F-test across the two main treatment periods will estimate whether there is any treatment effect and an F-test between the treatment period contrasts will estimate whether there is an order effect across subjects.

Individuals who withdraw from sildenafil or placebo treatment phases will be excluded from the primary analysis, although participants with adverse events who can not complete three weeks of treatment may be included if a physiological assessment can be performed after a minimum of 7 days of medication, including three doses within the last 24 hours. Participants without cilostazol exposure will be included in analyses directly comparing sildenafil and placebo, but not analyses comparing all the stages of treatment.

### Sample size calculations

At a power level of 0.9, with a 2-sided significance of 5%, a clinically relevant 0.12 unit change in pulsatility index (equivalent to a ∼20% difference in risk of recurrent stroke), and conservatively allowing for a standard deviation of differences in PI between repeated measures of 0.2, gives an estimated minimum sample size of 32 patients (paired t-test). Allowing for a 15% drop-out rate, 38 patients would be required. A sample size of 66 achieves 90% power to detect the non-inferiority of sildenafil compared with cilostazol using a non-inferiority margin of 0.08 (and mean of paired differences 0) at α = 0.025 (for a 95% CI) with a within-subject variance of 0.02. This equates to 75 patients in total with a 12% drop out rate.

A two-sided 5% significance level will be used for all hypothesis tests.

### Trial and data management

A Data and Safety Monitoring Board will review safety and recruitment. Annual reviews will begin 12 months after initiation or the first visit of the first patient (whichever is sooner), or at the request of the sponsor or CI. If a decision to terminate the study is contemplated, this will be discussed with the CI and the sponsor prior to a final decision being made. An independent study monitor will review the study on an annual basis.

The advisory futility threshold is set at a projected recruitment of <35 patients in 2 years (allowing for unavoidable delays due to the COVID-19 pandemic). Given the limited study size, no threshold is defined for efficacy for study cessation. Any statistically significant excess of SAEs in the treatment arm, or occurrence of unexpected treatment-related SAEs of sufficient severity (in the view of the DSMB or sponsor), may also result in early cessation of the study. If after 12 months the recruitment falls below the rate expected to complete the study in 3 years, the secondary comparison with cilostazol may be removed, and the overall study size reduced to 50 participants. Any data collected to that date will be retained and analysed according to the original study plan.

## Discussion and conclusions

This is the first study to directly compare the effects of phosphodiesterase inhibitors on cerebral pulsatility and reactivity in patients with cerebrovascular events and small vessel disease. Both pulsatility and reactivity are associated with cerebral small vessel disease in cohort studies, with plausible biological mechanisms for a causative effect. Previous trials have demonstrated both a clinical benefit of phosphodiesterase inhibition-dependent vasodilating medications on stroke risk (CSPS,^12^ ESPS2^22^) and cerebral small vessel disease, with parallel effects on cerebral pulsatility (ECLIPSE^23^) and reactivity (LACI 1^21^) If there is a significant physiological effect of sildenafil (or cilostazol) on these indices, this would support assessing the effect of these medications in longer-term clinical studies to determine their efficacy in prevention of progression of cerebral small vessel disease, chronic cognitive impairment, functional decline and recurrent stroke.
